# Lung Xenotransplantation Advances in the Context of Clinical Pandora's Box—Positioned Amid Innovations in Heart, Kidney, and Liver Xenografts

**DOI:** 10.1002/hcs2.70036

**Published:** 2025-10-09

**Authors:** Björn Nashan

**Affiliations:** ^1^ Research Centre of Big Data and Artificial Intelligence of Medicine The First Affiliated Hospital of Sun Yat‐Sen University Guangzhou China; ^2^ The Transplantation Center, First Affiliated Hospital, School of Life Sciences and Medical Center University of Sciences & Technology of China Hefei China

The milestone reported by He et al.—demonstrating pig‐to‐human lung xenotransplantation in a brain‐dead recipient—is a seminal moment in organ xenografting. It extends the horizons of xenotransplantation beyond the breakthroughs achieved in porcine heart and kidney grafts and aligns with the emergent clinical data on porcine liver applications [1].

Pioneering porcine heart transplants into terminal human recipients demonstrated that genetic engineering and immunosuppression could transiently forestall immune rejection [2, 3], though the patients finally did not survive for a number of reasons related to infection. Likewise, porcine kidney grafts functioned for several days post‐transplant in brain‐dead human models [4]. Out of the four clinical cases transplanted so far, only one is reported to still function [5].

Recent forays into porcine liver xenotransplantation mark a new frontier. At Anhui Medical University, surgeons successfully performed a heterotopic auxiliary transplant of a gene‐edited pig liver into a living human liver cancer patient (2024) [6]; the graft produced bile and demonstrated function in vivo without acute rejection. Similarly, at Xijing Hospital, a genetically modified porcine liver was implanted into a brain‐dead patient and maintained function for 10 days [7, 8]. These short‐term successes, while preliminary, emphasize the growing momentum in xenotransplant research [9, 10].

He et al. used lungs from donors lacking α‑Gal, Neu5Gc, and Sda antigens, alongside human complement regulators like CD46 and CD55 [1]. These modifications mirror those used in liver and renal models and underscore the importance of extensive glyco‐immunological tuning [10]. The Anhui auxiliary liver case similarly employed 10‐gene‐edited pigs with multiple human transgenes to temper immune activation [6].

Primary graft dysfunction (PGD) remains a leading challenge in lung transplantation due to the organ's delicate microvascular network. He et al. reported stable early lung function post‐transplant, though longer‐term studies remain necessary [1]. In the liver context, thrombocytopenia and coagulopathy are prominent, driven by porcine vWF and cross‐species incompatibility. Strategies integrating human thrombomodulin and complement inhibitors have partially mitigated this in preclinical models, but effective durability remains elusive [10].

Anti‐CD40 monoclonal antibody use in He et al.'s protocol aligns with emerging preferences for targeted costimulation blockade in xenografts. This contrasts with earlier reliance on anti‐CD154, which was associated with thrombosis [11, 12]. These strategies echo liver xenograft protocols, which have begun using tacrolimus, steroids, and B‐cell depletion with preliminary success and no acute rejection in the reported Chinese auxiliary and full size liver transplant case [6, 7].

While achievements in heart, kidney, lung, and auxiliary/full size liver xenografts are promising, translation into clinical protocols demands cautious progression, and global regulatory frameworks [13, 14, 15, 16]. Key next steps include:

1. Extended Survival Studies: Multiday lung xenotransplants in brain‐dead models or NHPs will provide crucial data on durability and PGD prevention.
2. Heuristic Immune Monitoring: Profiling antibody responses, complement activity, and cellular immunity must guide regimen optimization.
3. Organ‐Specific Protocol Standardization: Establishing minimal effective genetic edits and immunosuppression regimes across organ types will enhance reproducibility.
4. Ethical, Regulatory Frameworks: Longitudinal follow‐up for zoonoses (e.g., PERV) and transparent compassionate‐use criteria are prerequisites for clinical translation. Clinical auxiliary liver cases raise hope for bridging strategies but require governance benchmarks [15, 16].

He et al.'s pig lung xenotransplant in a human context is an incisive breakthrough that resonates with parallel advances in liver, heart, and kidney xenografting [1, 2, 3, 5, 6]. Collectively, these developments affirm the growing plausibility of porcine organs in clinical transplantation—albeit after rigorous, stepwise validation [8, 14, 15, 16]. The road toward clinical application must integrate immunobiology, organ‐specific challenges, and ethical safeguards in equal measure. Still, the Pandora's box opened by clinical application must be tempered with scientific prudence.

## Author Contributions

**Björn Nashan:** conceptualization, methodology, writing – review and editing, writing – original draft, validation.

## Ethics Statement

The author has nothing to report.

## Consent

The author has nothing to report.

## Conflicts of Interest

Professor Björn Nashan is a member of the *Health Care Science* Editorial Board. To minimize bias, he was excluded from all editorial decision‐making related to the acceptance of this article for publication.

## Data Availability

The author has nothing to report.
